# Preclinical Evaluation of Novel Tyrosine-Kinase Inhibitors in Medullary Thyroid Cancer

**DOI:** 10.3390/cancers14184442

**Published:** 2022-09-13

**Authors:** Davide Saronni, Germano Gaudenzi, Alessandra Dicitore, Silvia Carra, Maria Celeste Cantone, Maria Orietta Borghi, Andrea Barbieri, Luca Mignani, Leo J. Hofland, Luca Persani, Giovanni Vitale

**Affiliations:** 1 PhD Program in Experimental Medicine, University of Milan, 20100 Milan, Italy; 2 Department of Medical Biotechnology and Translational Medicine, University of Milan, 20100 Milan, Italy; 3 Laboratory of Geriatric and Oncologic Neuroendocrinology Research, Istituto Auxologico Italiano IRCCS, 20100 Milan, Italy; 4 Laboratory of Endocrine and Metabolic Research, Istituto Auxologico Italiano IRCCS, 20100 Milan, Italy; 5 Experimental Laboratory of Immuno-Rheumatology, Istituto Auxologico Italiano IRCCS, 20100 Milan, Italy; 6 Department of Clinical Sciences and Community Health, University of Milan, 20100 Milan, Italy; 7 Department of Molecular and Translational Medicine, University of Brescia, 25121 Brescia, Italy; 8 Department of Internal Medicine, Division of Endocrinology, Erasmus MC, 3015 GD Rotterdam, The Netherlands

**Keywords:** medullary thyroid cancer, tyrosine kinase inhibitors, apoptosis, migration, angiogenesis, zebrafish, tumor xenograft

## Abstract

**Simple Summary:**

Medullary thyroid carcinoma (MTC) is a neuroendocrine tumor arising from parafollicular calcitonin-secreting C cells of the thyroid. Most of the patients affected by MTC, especially the familial form, harbor a mutation of the RET proto-oncogene. In patients with advanced disease, medical therapy is represented by two tyrosine-kinase inhibitors: cabozantinib and vandetanib. However, their usage is limited by several adverse events and drug-resistance onset. The aim of this preclinical study was to evaluate the antitumor activity of novel molecules for the therapy of MTC: SU5402, an inhibitor of the fibroblast growth factor receptor type 1 (FGFR-1) and vascular endothelial growth factor receptor (VEGFR)-2; sulfatinib, a multi-target kinase inhibitor selective for FGFR-1 and the VEGFR-1, -2, and -3; SPP86, a RET-specific inhibitor. Our results suggest a potential role in targeting the FGFR and VEGFR signaling pathways as an alternative strategy for resistant tumors and a significative antitumor activity of this new RET-specific inhibitor.

**Abstract:**

Medullary thyroid carcinoma (MTC) is a neuroendocrine tumor arising from parafollicular C cells of the thyroid gland. In this preclinical study, we tested three tyrosine-kinase inhibitors (TKIs): SU5402, a selective inhibitor of fibroblast growth factor receptor (FGFR)-1 and vascular endothelial growth factor receptor (VEGFR)-2; sulfatinib, an inhibitor of FGFR-1 and VEGFR-1, -2, -3; and SPP86, a RET-specific inhibitor. The effects of these compounds were evaluated in vitro in two human MTC cell lines (TT and MZ-CRC-1), and in vivo using xenografts of MTC cells in zebrafish embryos. SU5402, sulfatinib and SPP86 decreased cell viability. Sulfatinib and SPP86 significantly induced apoptosis in both cell lines. Sulfatinib and SPP86 inhibited the migration of TT and MZCRC-1 cells, while SU5402 was able to inhibit migration only in TT cells. In vivo we observed a significant reduction in TT cell-induced angiogenesis in zebrafish embryos after incubation with sulfatinib and SPP86. In conclusion, sulfatinib and SPP86 displayed a relevant antitumor activity both in vitro and in vivo. Moreover, this work suggests the potential utility of targeting FGFR and VEGFR signaling pathways as an alternative therapy for MTC.

## 1. Introduction

Medullary thyroid carcinoma (MTC) is a neuroendocrine tumor arising from the calcitonin-producing parafollicular C cells of the thyroid [1]. In 75% of cases, MTC occurs sporadically, and in a familial form for the remaining 25%. Over 95% of patients with the hereditary form carry a gain of function, germline mutation of the *RET* proto-oncogene. These mutations lead to multiple endocrine neoplasia (MEN) type 2 syndromes (MEN2A and MEN2B) and isolated familial MTC. Sporadic MTC shows somatic mutations of the *RET* proto-oncogene in about 40–60% of patients [2,3,4]. The *RET* gene encodes a receptor tyrosine kinase able to modulate cell proliferation, survival, migration and differentiation. In MEN2A syndrome, the most common mutation of *RET* is a cysteine substitution at codon 634, while in MEN2B over 90% of all mutations occur in codon 918 (threonine replacing a methionine). In both cases, these mutations lead to a constitutive activation of the receptor, which is causative of the syndromes [5].

Surgery is the mainstay of treatment for MTC. However, this approach is not curative in the presence of metastasis. In these cases, external radiotherapy and/or chemotherapy play a marginal role [6,7], while somatostatin analogues are mainly able to control neuroendocrine symptoms [8,9]. Two tyrosine-kinase inhibitors (TKIs) are currently used as a first-line treatment of symptomatic MTC with unresectable, locally advanced or metastatic disease: cabozantinib, a potent inhibitor of RET, vascular endothelial growth factor receptor (VEGFR)-2 and c-Met [10,11,12]; and vandetanib, targeting RET, VEGFR-2/3 and epidermal growth factor receptor (EGFR) [13,14]. These drugs significantly increase progression-free survival. Unfortunately, not all patients respond to this therapy or can develop resistance due to the activation of alternative survival pathways [6,15]. In addition, long-term treatment is limited by several adverse events [16,17]. Indeed, the discontinuity of drug administration due to either disease progression or toxicity has been reported in about 40–55% of patients [18]. Therefore, new therapeutic strategies are urgently required. Pharmaceutical research is currently focusing on the development of new TKIs targeting alternative pathways and RET-specific inhibitors with potential clinical implications in patients with *RET*-negative MTC and *RET* mutation-positive MTC, respectively.

In light of these arguments, the aim of this preclinical study was to investigate the efficacy of novel TKIs in MTC: SU5402, a selective inhibitor for fibroblast growth factor receptor type 1 (FGFR-1) and VEGFR-2 [19,20,21]; sulfatinib, targeting FGFR-1 and VEGFR-1/2/3; and SPP86, a RET-specific inhibitor. Our experiments were conducted both in vitro through two human MTC cell lines (TT and MZ-CRC-1), and in vivo using an innovative zebrafish (*Danio rerio*) model for thyroid cancer [22,23].

## 2. Materials and Methods

### 2.1. Cell Line Culture

The human MTC cell lines, TT and MZ-CRC-1 characterized by C634W and M918T *RET* mutations, respectively, were kindly provided by Prof. Lips (University of Utrecht, The Netherlands) [24,25]. F-12 with Kaighn’s modification medium containing 10% fetal bovine serum, 2 mM glutamine, and 10^5^ U/I penicillin-streptomycin was used to culture cell lines in a humified atmosphere of 5% CO_2_. MTC cells were grown in 75 cm^2^ flasks and passaged once every 7 days by splitting 1:3.

### 2.2. RNA Isolation

Total RNA was extracted from TT and MZ-CRC-1 with trizol (Invitrogen, Waltham, CA, USA) according to the manufacturer’s instructions. RNA samples were store at −80 °C. Complementary DNA (cDNA) was reverse-transcribed using 2 µg of total RNA using GoScript™ Reverse Transcription System (cat. A5000, Promega Corporation, Madison, WI, USA) following the manufacturer’s instructions. 

### 2.3. Polymerase Chain Reaction (PCR)

PCR was performed to evaluate the expression of *FGFR-1*, *-2*, *-3* and *-4* and *fibroblast growth factor (FGF)-2* and *-8* in TT and MZ-CRC-1 cells. Each PCR reaction was carried out in a final volume of 25 μL using GoTaq^®^ G2 DNA Polymerase (M784B, Promega Corporation, Madison, WI, USA) according to manufacturer’s indications (5 μL of 5X reaction buffer with MgCl_2_, 1 μL of 10 mM dNTPs, 1 μL of 10 pmol/μL forward primer, 1 μL of 10 pmol/μL reverse primer, 1 μL of cDNA sample and 0.25 μL of 5 U/μL GoTaq^®^ G2 DNA Polymerase). The PCR protocol consisted in an initial denaturation step (5 min, 94 °C), followed by 35 cycles of amplification and a final 7 min extension step. Annealing temperatures were set to 60 °C for the FGFR-1 primer pair and 56°C for the remaining primer pairs. Water was used as negative control. PCR products were visualized using Midori Green Advanced staining (MG04, Nippon Genetics Europe) upon 2% agarose gel electrophoresis.

The primer sequences and the expected length of each amplified fragment are resumed in Table 1. All primers were synthesized by Eurofins Scientific (Milan, Italy).

### 2.4. Drug Preparation

SU5402 was obtained by Sigma-Aldrich (St. Louis, MO, USA), while both Sulfatinib and SPP86 were purchased by MedChemExpress (Monmouth Junction, NJ 08852, USA). All compounds were dissolved in DMSO. 

### 2.5. Cell Viability Assay

Cells were plated in 96 well plates at a density of 3 × 10^4^ cells per well. The day after, cell culture medium was replaced with medium containing different concentrations (ranging from 0.05 to 30 µM) of the drugs, or medium with equivalent DMSO concentration (vehicle) used as control. After 3 days, the medium was replaced, and the treatment was repeated. After 6 days, cell viability was analyzed using 3-(4,5-dymethylthiazol-2-yl)-2,5-dyiphenyltetrazolium bromide (MTT) assay, as previously described [24]. All experiments were performed in six replicates.

In vitro experiments (analysis of cell viability, cell cycle and apoptosis) were monitored for up to 6 days of drug incubation. We performed long-term treatments due to the slow doubling time (about 4 days) of the MTC cell lines, as previously reported [26]. Indeed, the effects of tested TKIs on cell viability were moderate after 3 days of incubation, as shown in Figure S1.

### 2.6. Cell Cycle Analysis

TT and MZ-CRC-1 were seeded in duplicates in six-well plates at the density of 2 × 10^5^ cells per well. The day after, cell culture medium was replaced with medium containing the EC_50_ concentration of each inhibitor (Table 2) or drug vehicle as control. After 3 days, the medium was replaced with a fresh one containing compounds at the EC_50_ concentrations for a further 3 days. At the end of the experiment, cells were harvested by gentle trypsinization, washed three times with cold PBS (calcium and magnesium free), and collected by centrifugation at 1200× *g* for 5 min. The pellets were resuspended and directly stained with propidium iodide (PI) (Sigma-Aldrich, St. Louis, MO, USA). Flow cytometric analysis was performed using a FACSCalibur instrument (BD Bioscience, San Jose, CA, USA) and CellQuest software, as previously described [25].

### 2.7. Flow Cytometric Analysis of Apoptosis

Cells were plated in duplicates in six-well plates at the density of 2 × 10^5^ cells per well. Cells were treated for 6 days, as previously described in the section of cell cycle analysis. On the sixth day, cells were harvested by gentle trypsinization, washed three times with cold PBS (calcium and magnesium free), and collected by centrifugation at 1200× *g* for 5 min. Pellets were re-suspended in 1X binding buffer (0.1 M HEPES/NaOH, pH 7.4, 1.4 M NaCl, 25 mM CaCl2) and stained with 5 μL of annexin V-FITC (BD Pharmingen, San Diego, CA, USA) and 10 μL PI (50 μg/mL in PBS). After 20 min of incubation at room temperature in the dark, 400 µM of 1X binding buffer was added to each tube. Flow cytometric analysis was performed using a FACSCalibur instrument (BD Bioscience, San Jose, CA, USA) and CellQuest software, as previously described.

### 2.8. Wound-Healing Assay

MTC cell lines were seeded in 6-well plates in duplicate (10^6^ cells/well) and cultured until they reached 100% confluence and growth media was renewed as needed. After creating a monolayer, cells were scratched orthogonally from the bottom of each well using a 10 μL sterile micropipette tip. 

Cells were washed with PBS to remove cell debris then supplemented with growth medium without (control condition) or with sulfatinib, SPP86 or SU5402 at their EC_50_ concentration. We took pictures of the scratches at T0 and after 72 h of incubation with a TKI. Before performing these experiments, we tested the effect of the EC_50_ concentration on cell viability after 3 days of incubation with each drug. At this time point, TKIs did not show any relevant effect on cell survival. Images of defined wounds were acquired through Leica DM IRE 2 (Inverted Fluorescence Motorized Phase Contrast Microscope) using a 10X objective at different time point: right after the scratch (T0) and after 3 days according to the cell type (TF). 

The wound-healing area was measured with ImageJ software (National Institutes of Health, Bethesda, MD, USA). Results were reported as wound healing percentage using the equation:

% wound-healing = 100 × [1 − (wound area at TF/wound area at T0)]

For each plate, at least 3 randomly selected images were acquired. All experiments were independently carried out in triplicate.

### 2.9. In Vivo Assay for Tumor-Induced Angiogenesis

We used a zebrafish transplantable model based on the implantation of neuroendocrine tumors in *Tg(fli1a:EGFP)^y1^* transgenic embryos [22,26,27]. Embryo and adult zebrafish were raised and maintained according to Italian (D.Lgs 26/2014) and European laws (2010/63/EU and 86/609/EEC). At 48 h post fertilization (hpf), embryos were anesthetized with 0.016% tricaine (Ethyl 3-aminobenzoatemethanesulfonate salt, Sigma-Aldrich^®^ Merck KGaA) and implanted with TT cells as previously described [27]. TT cells were labeled with a red fluorescent viable dye (CellTrackerTM CM-DiI dye, Invitrogen), following manufacturer’s instructions, resuspended with PBS and grafted into the subperidermal space of *Tg(fli1a: EGFP)^y1^* embryos, close to the sub-intestinal vein (SIV) plexus. After implantation, correctly grafted embryos were selected and were treated for 24 h with the drugs, directly dissolved into the fish water. The drug concentrations (0.25 µM and 2.5 µM) were identified on the basis of preliminary pharmacological experiments on *Tg(fli1a:EGFP)^y1^* embryos without tumor xenograft, aimed to detect the toxicity range for each compound, limiting the presence of morphological abnormalities. As untreated controls we considered injected embryos incubated in the fish medium and the vehicle in which the experimental substance was dissolved (DMSO). All implanted embryos were raised at 32 °C, a compromise temperature between 28 °C, optimal for zebrafish maintenance, and 37 °C, optimal for mammalian cell growth and metabolism. Tumor-induced angiogenesis was monitored in vivo by means of an epifluorescence microscope (Leica M205FA equipped with a Leica DFC450C digital camera; Leica, Wetzlar, Germany) and all images were taken after 24 h of treatment. As an arbitrary unit of tumor-induced angiogenesis, we calculated the total cumulative length of vessels sprouting from the SIV plexus and the common cardinal vein in each embryo by using Fiji software. Data were normalized against the mean of the control (DMSO), arbitrarily set to 1.0. The values reported in the graphs represent the mean ± standard error of the mean (S.E.M). In vivo experiments were performed with only TT cells, since MZ-CRC-1 induced a less potent stimulation of angiogenesis in zebrafish embryos.

### 2.10. Statistical Analysis

All experiments were carried out at least three times and gave comparable results. GraphPadPrism 5.0 (GraphPad Software, San Diego, CA, USA) was used for statistical analysis. The comparative statistical evaluation among groups was first performed by the ANOVA test. When significant differences were found, a comparison between groups was made using the Newman–Keuls test. In all analyses, values of *p* < 0.05 were considered statistically significant. The values reported in figures are the mean ± S.E.M.

## 3. Results

### 3.1. Characterization of FGF System in MTC Cells

**Figure 1:**
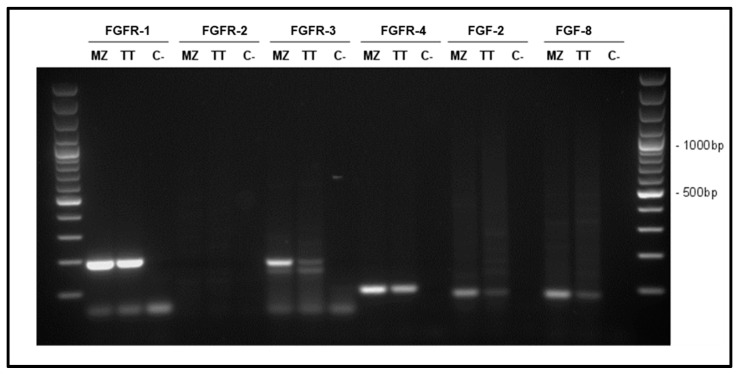
Representative results of *FGFR-1* (192 bp), *FGFR-2* (144 bp), *FGFR-3* (181 bp), *FGFR-4* (94 bp), *FGF-2* (83 bp) and *FGF-8* (82 bp) mRNA expression in TT and MZ-CRC-1 cells (MZ). Water was used as negative control (C).

We assessed the expression of *FGFR-1*, *FGFR-2*, *FGFR-3*, *FGFR-4*, *FGF-2* and *FGF-8* in TT and MZ-CRC-1 cells by PCR (Figure 1). In both cell lines, we observed a high signal for *FGFR-1* and expression of *FGFR-3* and *FGFR-4*. No expression for *FGFR-2* was observed. *FGF-2* and *FGF-8* were expressed in both cell lines.

### 3.2. Effects of TKIs on Cell Viability

**Figure 2:**
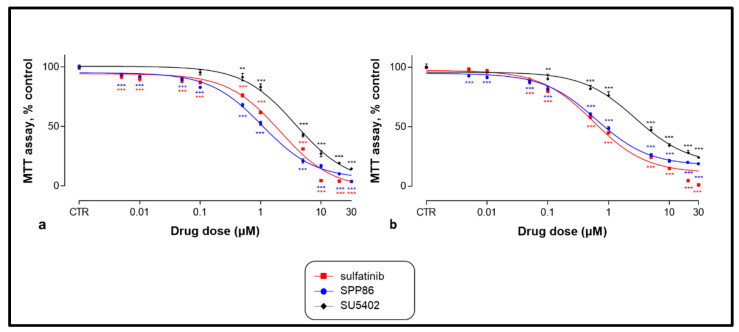
Dose-dependent effect of SU5402, sulfatinib and SPP86 on cell viability of TT (**a**) and MZ-CRC-1 (**b**) cell lines, as measured by the MTT assay. Cells were incubated for 6 days with vehicle (control) or with the drug at different concentrations, as described in Material and Methods. Dose response curves were expressed as nonlinear regression (curve fit) of log (concentration drug) versus the percentage of control. Values represent the mean and standard error of the mean of at least three independent experiments in six replicates. **: *p* < 0.01, ***: *p* < 0.001, CTR: control, MTT: 3-(4,5-dymethylthiazol-2-yl)-2,5-diphenyltetrazolium bromide.

After 6 days of incubation, while untreated cells showed no sign of suffering, SU5402, sulfatinib and SPP86 significantly inhibited cell viability of both MTC cell lines in a dose-dependent manner (Figure 2). Table 2 reports for each drug the EC_50_ concentration and the maximal inhibitory effect. In TT cells (Figure 2a and Table 2), the EC_50_ of all three molecules were significantly different (*p* < 0.001), with SPP86 presenting the lowest EC_50_, while no statistically significant difference was observed between the values of maximal inhibition of each TKI. In MZ-CRC-1 cells (Figure 2b and Table 2), the EC_50_ of SPP86 and sulfatinib were significantly lower than SU5402 (*p* < 0.001). Maximal inhibition of proliferation after sulfatinib was higher than SPP86 (*p* < 0.05) and SU5402 (*p* < 0.01), while no difference between SSP86 and SU5402 was observed. For further in vitro experiments, we selected the EC_50_ concentration for each drug.

### 3.3. Effects of TKIs on Cell Cycle

**Figure 3:**
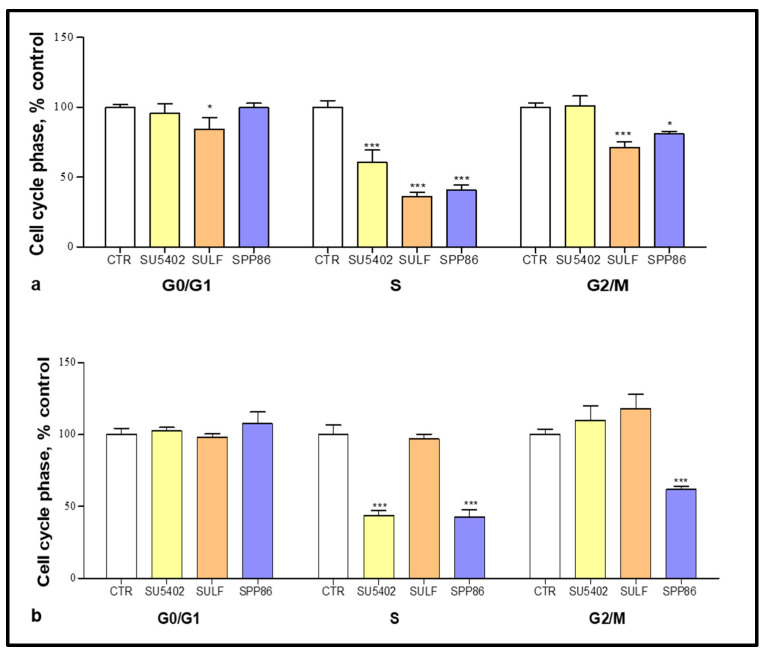
Cell cycle analysis after 6 days of incubation with SU5402, sulfatinib (SULF) and SPP86 in TT (**a**) and MZ-CRC-1 (**b**) cell lines. Cells were detected by FACS analysis after staining with propidium iodide. CTR values have been set to 100%. Values represent the mean ± standard error of the mean of at least 3 independent experiments. *: *p* < 0.05, ***: *p* < 0.001, CTR: control, SULF: sulfatinib.

Cell cycle phase distribution was evaluated by FACS analysis after incubating TT and MZ-CRC-1 cells with several TKIs. In TT cells (Figure 3a), all compounds significantly decreased the number of cells in S phase, with sulfatinib and SPP86 showing a higher effect compared to SU5402 (−63.7%, −59.2% and −39.4%, respectively, *p* < 0.001 for all TKIs vs. control). Sulfatinib and SPP86 also reduced the fraction of cells in G2/M phase (−28.7%, *p* < 0.01; −18.9%, *p* < 0.05, respectively) in comparison with control. In MZ-CRC-1 cells (Figure 3b), the S phase was significantly impaired after SU5402 and SPP86 (−56.3% and −57.2%, respectively, both *p* < 0.001 vs. control), with the latter having a relevant effect also in the G_2_/M phase (−38%, *p* < 0.001 vs. control). Representative experiments were reported in Figures S2 and S3. These data are indicative of cell cycle perturbation, particularly after treatment with sulfatinib and SPP86. These effects are probably secondary to the ability of these compounds to stimulate apoptosis and/or necrosis, as suggested by the increase in cells in the subG1 area after the treatment compared to control (Figures S2 and S3).

### 3.4. Effects of TKIs on Apoptosis and Necrosis

**Figure 4:**
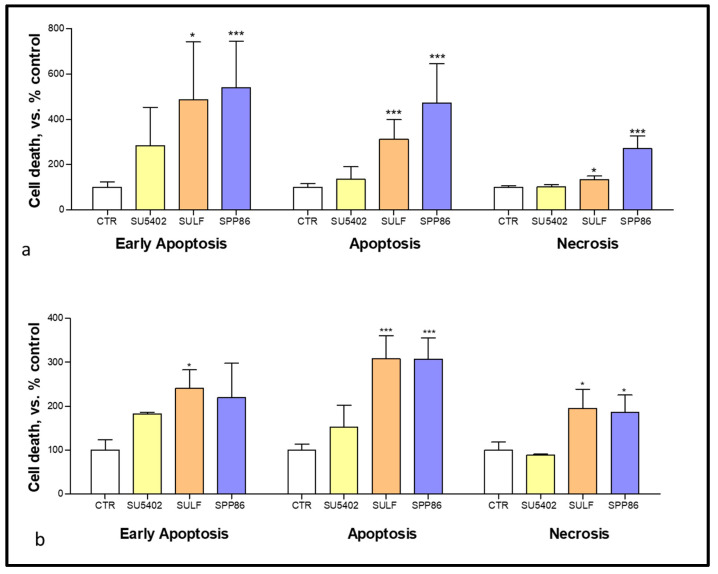
Modulation of cell death analysis after 6 days of incubation with SU5402, SULF and SPP86 in TT (**a**) and MZ-CRC-1 (**b**) cell lines through flow cytometry with annexin V and propidium iodide. The proportions of cells in early apoptosis, late apoptosis and necrosis are expressed as percentage compared with CTR and represent the mean ± standard error of the mean of at least 3 independent experiments. *: *p* < 0.05, ***: *p* < 0.001, CTR: control, SULF: sulfatinib.

In order to evaluate the effects of these selected TKIs on both apoptosis and necrosis we performed flow cytometry with annexin V and PI after 6 days of treatment. In TT cells (Figure 4a) both sulfatinib and SPP86 significantly increased the fractions of cells in early and late apoptosis and necrosis (sulfatinib vs. control: +388%, *p* < 0.05; +212.2%, *p* < 0.001; +32.1%, *p* < 0.05, respectively; SPP86 vs. control: +440.2%; +370.8% and +170%, all *p* < 0.001, respectively). In MZ-CRC-1 cells (Figure 4b), sulfatinib increased all fractions of cells compared to control (early apoptosis +141.1%, *p* < 0.05; late apoptosis +208.3%, *p* < 0.001, necrosis +95.2%, *p* < 0.05), while SPP86 had a relevant impact on late apoptosis (+207.4%, *p* < 0.001) and necrosis (+85.9%, *p* < 0.05). Representative experiments were reported in Figures S4 and S5. These data suggested that both sulfatinib and SPP86 showed relevant pro-apoptotic and pro- necrotic activities in MTC cells.

### 3.5. Effects of TKIs on Cell Migration

**Figure 5:**
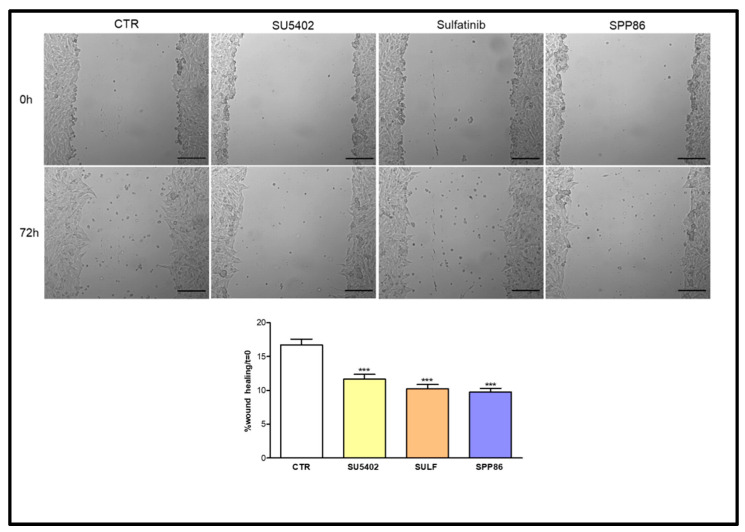
Effect of SU5402, SULF and SPP86 on TT cell migration compared to vehicle treated CTR. The area of wound was recorded at 0 and 3 days, and the percentage of wound healing with respect to T0 was calculated using the equation reported in Material and Methods section. Data are reported as mean ± standard error of the mean of at least 3 independent experiments. Scale bar 200 μm. ***: *p* < 0.001, CTR: control, SULF: sulfatinib.

We investigated the impact in vitro of TKIs on cell migration through a wound-healing assay. In TT cells (Figure 5) SPP86 had the most relevant inhibitory effect on cell migration (−41.6% vs. control, *p* < 0.001), followed by sulfatinib (−38.5% vs. control, *p* < 0.001) and SU5402 (−30.3% vs. control, *p* < 0.001).

**Figure 6:**
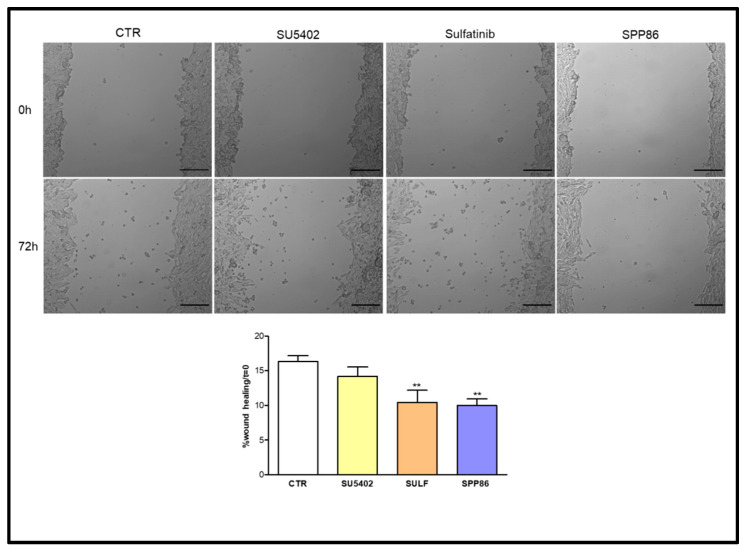
Effect of SU5402, SULF and SPP86 on MZ-CRC-1 cell migration compared to vehicle treated CTR. The area of wound was recorded at 0 and 3 days, and the percentage of wound healing with respect to T0 was calculated using the equation reported in the Material and Methods section. Data were reported as mean ± standard error of the mean of at least 3 independent experiments. Scale bar 200 μm. **: *p* < 0.01, CTR: control, SULF: sulfatinib.

In MZ-CRC-1 cells (Figure 6), both sulfatinib and SPP86 inhibited cell migration compared to control (−36.1% and −38.6%, respectively, both *p* < 0.01). While SU5402 was not able to significantly affect the wound-healing process.

### 3.6. Effects of TKIs on TT Cell Line-Induced Angiogenesis

**Figure 7:**
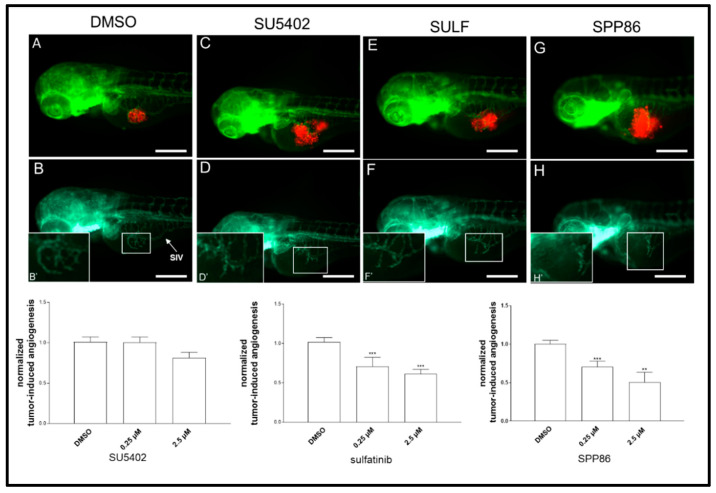
Effects of SU5402, sulfatinib and SPP86 on TT cell-induced angiogenesis in zebrafish. There is a representative image of an injected and treated embryo for each inhibitor (2.5 µM) and control (vehicle DMSO) in panels (**A**,**C**,**E**,**G**). The red fluorescence channel, corresponding to red stained TT cells, was omitted in panels (**B**,**D**,**F**,**H**) to highlight the tumor-induced microvascular network sprouting from the SIV (sub-intestinal vein) plexus (white arrow). Digital magnifications of the graft region are shown in white-boxed regions (**B**’,**D**’,**F**’,**H**’). Graphs below report the results of tumor-induced angiogenesis quantification at 24 h post-injection. All images are oriented so that rostral is to the left and dorsal is at the top. Scale bar: 100 µm. **: *p* < 0.01, ***: *p* < 0.001.

To evaluate the antiangiogenic potential of these TKIs, we took advantage of an in vivo platform that we have recently developed implanting neuroendocrine tumor cells in *Tg(fli1a:EGFP)^y1^* zebrafish embryos [22,27,28]. Forty-eight hpf *Tg(fli1a:EGFP)^y1^* embryos were grafted with TT cells (Figure 7).

Subsequently, injected embryos were incubated with two concentrations (0.25 and 2.5 µM) of each compound or DMSO (control) dissolved in fish water. After 24 h of treatment, we evaluated the effects of TKIs on tumor-induced angiogenesis, following in vivo the formation of endothelial structures around the tumor implant in each experimental group. SU5402 lightly reduced the formation of novel vessels at high concentration (2.5 µM), although this effect was not statistically significant. On the other hand, sulfatinib and SPP86 displayed a significant and similar inhibition of TT-induced angiogenesis compared to controls at both 0.25 µM and 2.5 µM (Figure 7).

## 4. Discussion

MTC is a highly vascularized tumor. Indeed, angiogenesis plays a relevant role for the progression of this neoplasm. Patients with advanced MTC are currently treated with drugs targeting RET and other tyrosine kinases involved in angiogenesis [29]. To date 2 multikinase inhibitors (vandetanib and cabozantinib) and 2 RET selective inhibitors (selpercatinib and pralsetinib) have been approved by the Food and Drug Administration (FDA) for the treatment of advanced MTC [30].

In this preclinical study, we evaluated the potential antitumor activity of new TKIs in MTC, focusing on molecules targeting FGFR-1/VEGFR (SU5402, inhibitor of FGFR-1 and VEGFR-2; sulfatinib, inhibitor of FGFR-1 and VEGFR-1/2/3) and RET (SPP86, a RET-specific inhibitor).

SU5402, sulfatinib and SPP86 inhibited cell viability of both TT and MZ-CRC-1 cells in a dose-dependent manner. Both sulfatinib and SPP86 showed the most potent antiproliferative activity. Consistently, these two molecules shared a similar effect on cell death, and in stimulating apoptosis and necrosis in both cell lines. SPP86 appeared to be the most effective compound in affecting the cell cycle. It was able to decrease the percentage of TT and MZ-CRC-1 cells in S phase and in G_2_/M phase. Sulfatinib and SPP86 decreased the migration of both MTC cells in a wound healing assay, while SU5402 showed a significant inhibition only in TT cells. In vivo, sulfatinib and SPP86 were able to significantly decrease the tumor-induced angiogenesis of implanted TT cells in zebrafish embryos. Therefore, both SPP86 and sulfatinib showed the most relevant and promising antitumor activity in our in vitro and in vivo models for MTC.

SPP86 is a RET-specific inhibitor [31]. Most patients with MTC are characterized by activating mutations of the *RET* proto-oncogene [32,33], which makes this receptor a suitable target for MTC [34]. Currently, cabozantinib and vandetanib are the first-line therapy in patients with advanced MTC [35]. However, their long-term use is limited by toxicity due to the multi-target profile. Targeting molecular receptors such as VEGFRs, which are involved in many processes (angiogenesis, wound healing, mucosal integrity, and renal function), is associated with several adverse effects, such as hypertension, leucopenia, proteinuria, diarrhea, fatigue and anorexia [17,36,37,38]. The development of new molecules specifically targeting the RET receptor could be of high importance to limit the onset of adverse effects in MTC patients positive for somatic *RET* mutations. In the present preclinical study, we observed a relevant antitumor activity of SPP86 with a prospective clinical role in the therapy of patients with somatic *RET* mutations. SPP86, targeting RET with high selectivity, could potentially prevent several side-effects observed after the use of multi-target TKIs currently approved for MTC treatment (vandetanib and cabozantinib).

We have recently evaluated the effects of vandetanib and cabozantinib on TT and MZ-CRC-1 cells’ survival and tumor-induced angiogenesis, through the same in vitro and in vivo tools adopted in the present study [39]. Comparing these data with the results of the present study, in TT cells the maximal inhibition of cell viability after SPP86 (−100%), sulfatinib (−100%) and SU5402 (−97.3%) were higher than that of vandetanib (−92.7%, *p* < 0.001, for all drugs) and cabozantinib (−91.2%, *p* < 0.001, for all drugs); while in MZ-CRC-1 cells the maximal inhibition of SPP86 (−82.5%) and sulfatinib (−88.7%) were higher than that of cabozantinib (−74.9%, *p* < 0.01 and *p* < 0.001 respectively). Additionally, the anti-proliferative activities of vandetanib and sulfatinib were modulated by a relevant induction of apoptosis/necrosis. Moreover, at the concentration of 2.5 µM the impact of sulfatinib (−39%) and SPP86 (−49%) in inhibiting TT-induced angiogenesis was comparable with that observed after vandetanib (−37%) in zebrafish embryos, while they were significantly less potent than cabozantinib (−86.4%, *p* < 0.001 for sulfatinib and *p* < 0.05 for SPP86). In this model, the antiangiogenic activity of SU5402 (−18.6%) was relevantly less potent than vandetanib (−37%) and cabozantinib (−86.4%) (*p* < 0.05 and *p* < 0.01 respectively) [39].

Taking these considerations into account, SPP86 could potentially represent a valid alternative to standard therapy, as its antitumor activity appears to be comparable or even stronger than vandetanib in these preclinical studies, but probably with reduced side-effects due to the high selectivity for RET. Two RET-specific molecules have been recently approved by the FDA for the therapy of *RET*-mutant MTC: selpercatinib and pralsetinib [40,41,42,43]. Selpercatinib showed efficacy in an open-label, phase 1–2 trial that enrolled 55 patients with MTC that were previously treated with vandetanib or cabozantinib and 88 patients that did not received any previous treatment. In this study, 69% of the first group reached a response, while 73% of the second group had an objective response [44]. Treatment with selpercatinib was well tolerated with mainly low-grade toxic effects [44] also in children [45]. In another phase 1–2 study, pralsetinib was tested in patients affected by *RET*-mutated thyroid cancer (both with MTC or with *RET* fusion–positive thyroid cancer). Pralsetinib showed a good efficacy and was well-tolerated. An overall response has been reached in 33/55 (60%) of MTC patients, that previously received cabozantinib or vandetanib, with a median time to first response of 3.7 months. In 15 of 21 (71%) of treatment naïve MTC patients, an overall response has been observed within 5.6 months [46]. However, also for these selective RET inhibitors is possible the development of drug resistance, but the involved mechanisms are still unclear. Solomon et al. [47], analyzed the circulating tumor DNA of patients whose disease progressed after an initial response to selpercatinib. This study showed the onset of a *RET* G810 mutation that conferred resistance against selpercatinib. 

In the literature there are no clinical or preclinical studies on the antitumor activity of SPP86 in MTC. There are only few in vitro studies on this compound showing a high selectivity for RET (IC_50_ of 8 nM) and an ability to inhibit RET signaling in breast cancer (MCF-7) and papillary thyroid carcinoma (TPC-1) cell lines at low concentrations [31,48]. Our study supports the clinical attractiveness of using SPP86 in the treatment of patients with *RET*-positive MTC, considering the relevant anti-tumor activity of this molecule modulated by stimulation of apoptosis/necrosis and inhibition of migration and tumor-induced angiogenesis. However, it is not currently possible to determine whether SPP86 is more potent than selpercatinib and pralsetinib. It would be interesting to compare in the future the antitumor activity of SPP86 with selpercatinib and pralsetinib, including a detailed analysis on specific *RET*-mutations addressed by SPP86.

TKIs are cytostatic drugs able to slow or stop the growth of cancer cells, without killing them. For this reason, the treatment should be continued as long as there is evidence of clinical benefit. The onset of drug resistance is a main cause of therapeutic failure in patients with tumors [49]. In MTC, the majority of patients develop resistance after an initial response to vandetanib and cabozantinib, probably due to the activation of alternative survival pathways. Furthermore, another significant proportion of patients do not respond at all to the standard therapies [6,15]. It is then crucial to evaluate different pathways involved in tumor development and survival that could potentially represent novel pharmacological targets. The evaluation of the potential role of FGFR/VEGFR pathways in MTC treatment, as an alternative target to conventional drugs targeting RET could be interesting, particularly in patients with MTC negative for RET mutations. Our work provides hints on the effect of targeting both FGFR and VEGFR in MTC. These pathways are known to be relevant mitogenic signaling pathways activated during the onset of drug resistance in tumors [6,50,51,52]. The potential role of FGF and VEGF signaling pathways in carcinogenesis and tumor progression has been assessed in several neoplasms [53,54,55,56,57,58,59,60,61,62,63]. Although less studied in MTC, these pathways appear to be involved in thyroid cancer progression. Komorowski et al., [64] evaluated the blood concentrations of angiogenic growth factors (VEGF and FGF), matrix metalloproteinases and tissue inhibitors of matrix in 22 patients with thyroid cancers (three of them with MTC). While the blood concentration of VEGF was similar between control healthy subjects and patients with cancer, blood concentrations of FGF-2 were significantly higher in patients with thyroid cancer compared with controls (29.52 ± 4.99 vs. 6.05 ± 1.43 pg/mL; *p* < 0.001). Ezzat et al., showed increased expression of FGFR4 in TT cells [65]. Moreover, they provided evidence that the inhibition of FGFR4 phosphorylation arrested cell proliferation. Interestingly, Heilman et al. [66] observed amplifications of *FGF3* and *FGF19* genes in 9% of advanced MTC. In MTC, the overexpression of VEGFR-2 has been correlated to advanced tumor stage, as this receptor was expressed at higher levels in metastasis that in primary tumors [67]. In different clinical trials, multi-target TKIs targeting FGF and VEGF receptors have shown promising efficacy in MTC patients. Lenvatinib (targeting FGFR-1 -4, VEGFR-1 -3, RET, c-kit, PDGFRα and SCFR) showed a partial response rate in a phase II trial in 36% of patients, while the progression-free survival was 9 months [68]. A multicenter, open-label, phase II trial tested sulfatinib in 59 patients with advanced thyroid cancer, including 27 MTC. In MTC patients sulfatinib showed an objective response rate of 22.2%, with the majority (88%) achieving a disease control, and a progression-free survival of 11.1 months [69]. Anlotinib is a multi-targeting TKI with antitumor activity against FGFR-1 -3, VEGFR-1 -3, c-kit and PDGFRα. In a multicenter, randomized, double-blind phase IIB clinical trial in patients with locally advanced or metastatic MTC, anlotinib was able to lengthen the average progression-free survival from 11.1 months (placebo group) to 20.7 months. At the end of the study, 30 out of 62 patients reached partial response with an objective response rate in 48.2% of patients [70]. Most of these TKIs are designed to inhibit several pathways, including FGF/VEGF signaling. Therefore, it is difficult to extrapolate from these studies the potential antitumor activity in specifically blocking FGFRs and VEGFRs in MTC.

The present study supports the effectiveness of targeting FGFR/VEGFR pathways as an alternative strategy to impair MTC cell proliferation and progression. Indeed, both SU5402 and sulfatinib (TKIs without any relevant effect on RET) showed a potent antitumor activity modulated by the inhibition of these pathways. Although the use of immortalized cell lines with *RET* mutations may represent a limitation, these data provide a strong rationale to define in the future the potential antitumor activity of drugs targeting FGFR/VEGFR system particularly in patients with *RET*-negative MTC.

## 5. Conclusions

This study revealed a significant antitumor activity exerted by SPP86 in preclinical models of MTC, suggesting a good efficacy of this new specific RET inhibitor. In addition, the relevance of targeting the FGFR/VEGFR system, through sulfatinib and SU5402, suggests a potential role of these pathways in the therapy of MTC. 

## Figures and Tables

**Table 1:** Primer sequence, annealing temperature (Ta) and expected length of PCR product in base pair (bp).

Gene	Primer Type	Primer Sequence	Ta	Length
FGFR-1	Forward Reverse	GGGCTGGAATACTGCTACAA GCCAAAGTCTGCTATCTTCATC	60 °C	192 bp
FGFR-2	Forward Reverse	GGATAACAACACGCCTCTCTT GCCCAAAGCAACCTTCTC	56 °C	144 bp
FGFR-3	Forward Reverse	TGGTGTCCTGTGCCTACC CCGTTGGTCGTCTTCTTGT	56 °C	181 bp
FGFR-4	Forward Reverse	AACCGCATTGGAGGCATT TCTACCAGGCAGGTGTATGT	56 °C	98 bp
FGF-2	Forward Reverse	TGTGTCTATCAAAGGAGTGTG CCGTAACACATTTAGAAGCCA	56 °C	83 bp
FGF-8	Forward Reverse	TCTCCCAACAGCATGTGAG CTGTAGAGTTGGTAGGTCCG	56 °C	82 bp

**Table 2:** Growth inhibition of MTC cell lines after 6 days of incubation with TKIs.

Cell Line	TKI	EC_50_	Maximal Inhibition
	SU5402	3.6 µM	−96.5%
TT	Sulfatinib	2 µM	−100%
	SPP86	1.3 µM	−100%
	SU5402	2.6 µM	−80.6%
MZ-CRC-1	Sulfatinib	0.6 µM	−89.5%
	SPP86	0.6 µM	−82.5%

## Data Availability

The data presented in this study is available on request from the corresponding author.

## Supplementary Materials

The following supporting information can be downloaded at: https://www.mdpi.com/article/10.3390/cancers14184442/s1, Figure S1: Dose–dependent effects of SU5402, sulfatinib and SPP86 on cell viability of TT (**a**) and MZ-CRC-1 (**b**) cells after 3 days of incubation. *: *p* < 0.05, **: *p* < 0.01, ***: *p* < 0.001, CTR: control; Figure S2: Representative experiments of cell cycle analysis in TT cells after incubation with vehicle DMSO as control (CTR), SU5402, sulfatinib and SPP86. Percentages of cells in each cell cycle phase are reported; Figure S3: Representative experiments of cell cycle analysis in MZ-CRC-1 cells after incubation with vehicle DMSO as control (CTR), SU5402, sulfatinib and SPP86. Percentages of cells in each cell cycle phase are reported; Figure S4: Representative experiments of apoptosis, evaluated in TT cells through flow cytometry with annexin V and propidium iodide, after incubation with vehicle DMSO as control (CTR), SU5402, sulfatinib and SPP86. Percentages of cells in necrosis (N), apoptosis (A) and early apoptosis (EA) are reported; Figure S5: Representative experiments of apoptosis, evaluated in MZ-CRC-1 cells through flow cytometry with annexin V and propidium iodide, after incubation with vehicle DMSO as control (CTR), SU5402, sulfatinib and SPP86. Percentages of cells in necrosis (N), apoptosis (A) and early apoptosis (EA) are reported.
