# Supplementary material for: Preclinical Evaluation of Novel Tyrosine-Kinase Inhibitors in Medullary Thyroid Cancer
Source: Cancers (Basel). 2022 Sep 13;14(18):4442. doi: 10.3390/cancers14184442 (PMC9497079; doi:10.3390/cancers14184442)
Supplement: Supplementary file 1 [file cancers-14-04442-s001.zip › cancers-1882579-supplementary.pdf]

cancers

MDPI

Preclinical Evaluation of Novel Tyrosine-Kinase Inhibitors in Medullary Thyroid Cancer

Davide Saronni, Germano Gaudenzi, Alessandra Dicitore, Silvia Carra, Maria Celeste Cantone, Maria Orietta Borghi, Andrea Barbieri, Luca Mignani, Leo J. Hofland, Luca Persani and Giovanni Vitale

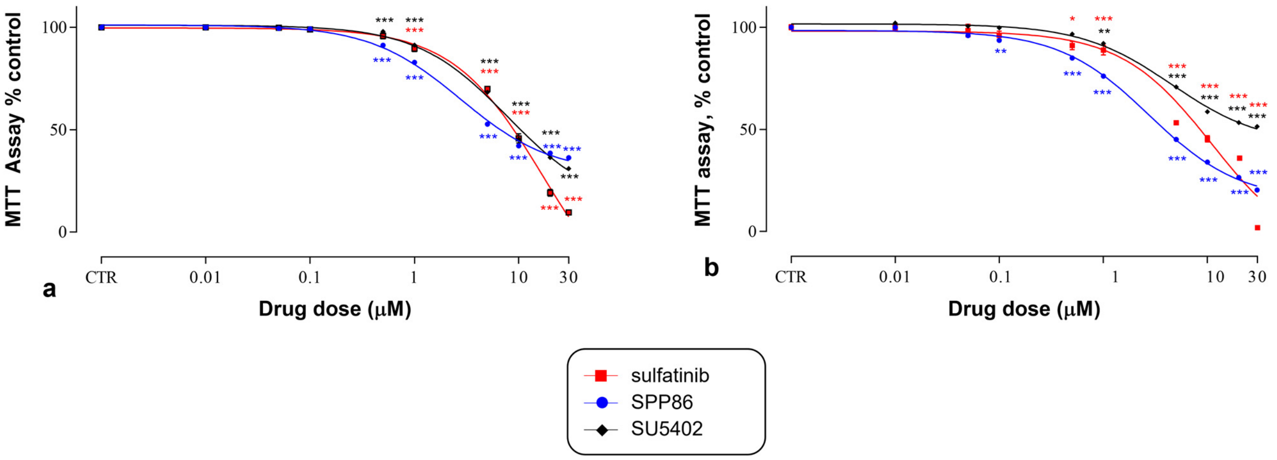

Figure S1. Dose-dependent effects of SU5402, sulfatinib and SPP86 on cell viability of TT (**a**) and MZ-CRC-1 (**b**) cells after 3 days of incubation. *: $p < 0.05$, **: $p < 0.01$, ***: $p < 0.001$, CTR: control.

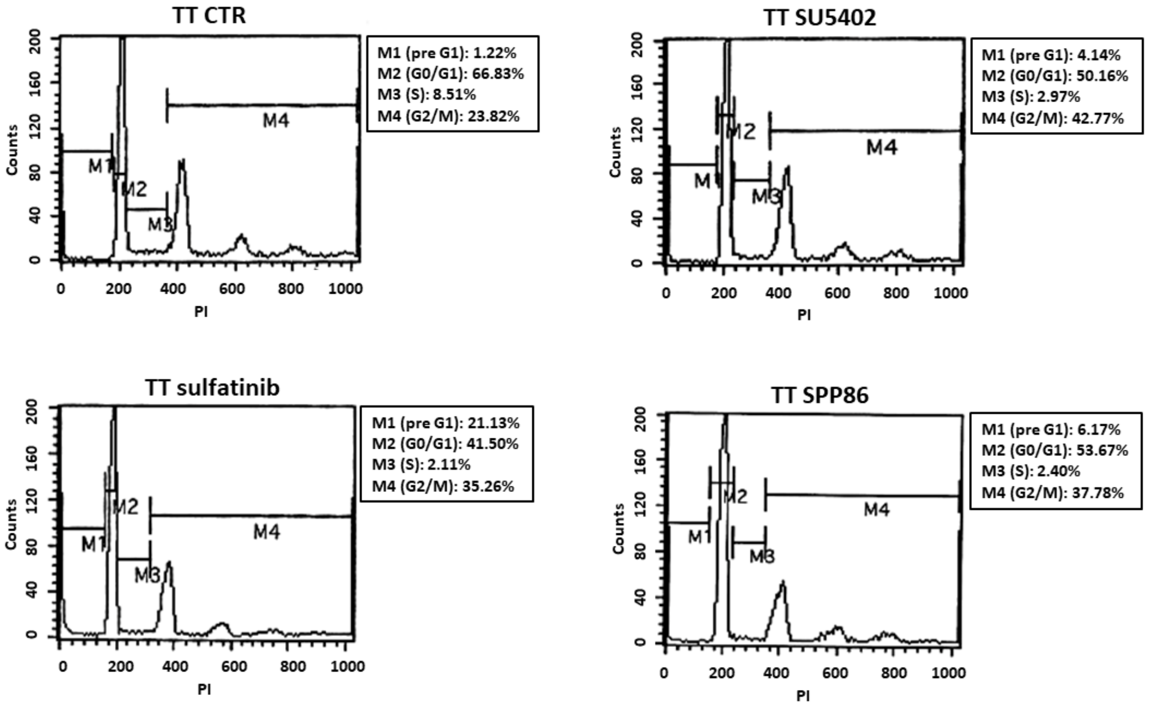

Figure S2. Representative experiments of cell cycle analysis in TT cells after incubation with vehicle DMSO as control (CTR), SU5402, sulfatinib and SPP86. Percentages of cells in each cell-cycle phase are reported.

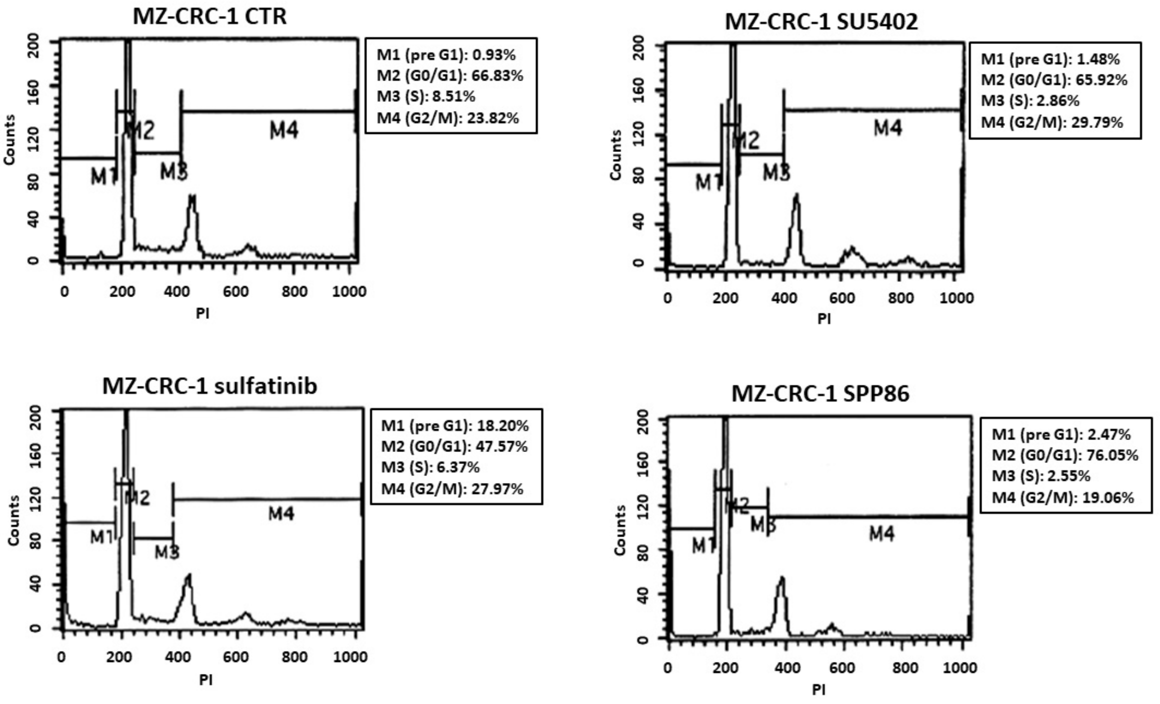

Figure S3. Representative experiments of cell cycle analysis in MZ-CRC-1 cells after incubation with vehicle DMSO as control (CTR), SU5402, sulfatinib and SPP86. Percentages of cells in each cell-cycle phase are reported.

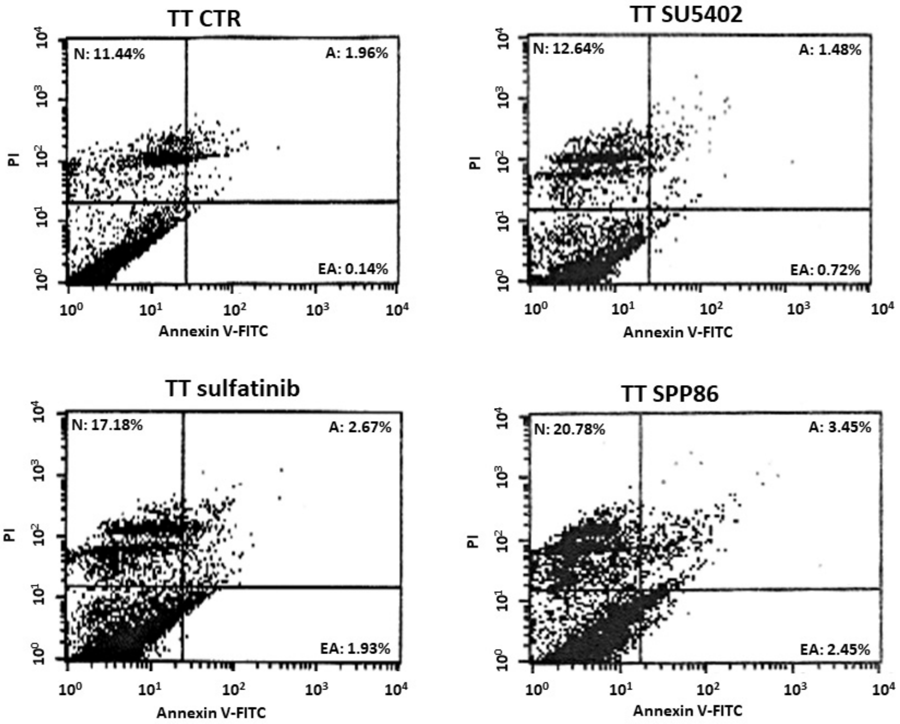

Figure S4. Representative experiments of apoptosis, evaluated in TT cells through flow cytometry with Annexin V and propidium iodide, after incubation with vehicle DMSO as control (CTR), SU5402, sulfatinib and SPP86. Percentages of cells in necrosis (N), apoptosis (A) and early apoptosis (EA) are reported.

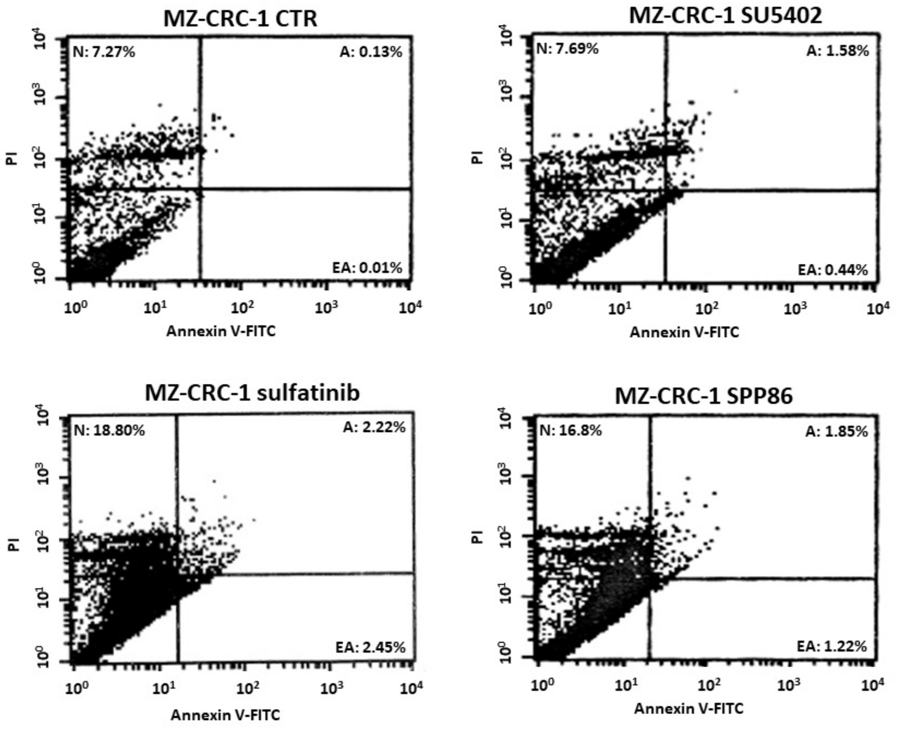

Figure S5. Representative experiments of apoptosis, evaluated in MZ-CRC-1 cells through flow cytometry with Annexin V and propidium iodide, after incubation with vehicle DMSO as control (CTR), SU5402, sulfatinib and SPP86. Percentages of cells in necrosis (N), apoptosis (A) and early apoptosis (EA) are reported.
